# Loss of Kex2 Affects the *Candida albicans* Cell Wall and Interaction with Innate Immune Cells

**DOI:** 10.3390/jof6020057

**Published:** 2020-04-29

**Authors:** Manuela Gómez-Gaviria, Nancy E. Lozoya-Pérez, Monika Staniszewska, Bernardo Franco, Gustavo A. Niño-Vega, Hector M. Mora-Montes

**Affiliations:** 1 Departamento de Biología, División de Ciencias Naturales y Exactas, Campus Guanajuato, Universidad de Guanajuato, Guanajuato Gto 36050, Mexico; manuela.gomezg8@gmail.com (M.G.-G.); nelppat@hotmail.com (N.E.L.-P.); bfranco@ugto.mx (B.F.); gustavo.nino@ugto.mx (G.A.N.-V.); 2 Faculty of Chemistry, Warsaw University of Technology, Noakowskiego 3, 00-664 Warsaw, Poland; mstaniszewska@ch.pw.edu.pl

**Keywords:** secretory pathway, protease, mannan, immune sensing, mannan, glycoproteins

## Abstract

The secretory pathway in *Candida albicans* involves the protein translocation into the lumen of the endoplasmic reticulum and transport to the Golgi complex, where proteins undergo posttranslational modifications, including glycosylation and proteolysis. The Golgi-resident Kex2 protease is involved in such processing and disruption of its encoding gene affected virulence and dimorphism. These previous studies were performed using cells without *URA3* or with *URA3* ectopically placed into the *KEX2* locus. Since these conditions are known to affect the cellular fitness and the host–fungus interaction, here we generated a *kex2*Δ null mutant strain with *URA3* placed into the neutral locus *RPS1*. The characterization of this strain showed defects in the cell wall composition, with a reduction in the *N*-linked mannan content, and the increment in the levels of *O*-linked mannans, chitin, and β-glucans. The defects in the mannan content are likely linked to changes in Golgi-resident enzymes, as the α-1,2-mannosyltransferase and α-1,6-mannosyltransferase activities were incremented and reduced, respectively. The mutant cells also showed reduced ability to stimulate cytokine production and phagocytosis by human mononuclear cells and macrophages, respectively. Collectively, these data showed that loss of Kex2 affected the cell wall composition, the protein glycosylation pathways, and interaction with innate immune cells.

## 1. Introduction

*Candida albicans* is one of the most important opportunistic fungal pathogens of humans, the leading etiological agent of candidiasis, and is associated with high morbidity and mortality rates, in particular in immunocompromised populations [1]. It belongs to the commensal microbiota of the mucosal surfaces and skin, colonizing the gastrointestinal and genitourinary tracts of healthy individuals [2]. However, when the local or systemic host defense mechanisms are temporally or permanently affected, this organism can cause life-threatening infections [3].

The *C. albicans* cell wall has been thoroughly studied in the last decade and it is well-established nowadays that it contributes to the maintenance of the cell integrity and participates in the interactions that the fungal cell establishes with the surrounding environment [4,5,6,7,8]. This is a stratified structure organized in two layers: the innermost, closer to the plasma membrane, is composed of the structural polysaccharides chitin, β-1,3-, and β-1,6-glucans; while the outermost layer is composed of proteins covalently modified with *N*-linked or *O*-linked mannans, which are commonly named as mannoproteins [5,6,9,10,11]. The *N*-linked mannans are highly branched oligosaccharides with mannose residues bound with different glycosidic linkages; while *O*-linked mannans are lineal and shorter oligosaccharides composed of up to seven α-1,2-mannose units [5,12]. Both the *N*-linked and *O*-linked mannans structures contribute to the immunological identity of *C. albicans* and are considered pathogen-associated molecular patterns that interact with pattern recognition receptors of the innate immune cells (PRRs). It has been reported that TLR4 recognizes *O*-linked mannans, while mannose receptors, DC-SIGN, mincle, dectin-2, and dectin-3 recognize the *N*-linked mannans [13,14,15,16,17,18].

The protein glycosylation pathway is responsible for the synthesis of *N*-linked and *O*-linked mannoproteins and belongs to the canonical protein secretory pathway [5]. This metabolic route begins in the endoplasmic reticulum, where both soluble and membrane-bound proteins undergo posttranslational modifications, including glycosylation and proteolytic cleavage [19]. Next, proteins are transported to the Golgi complex, where are subjected to further modifications, before being directed to the final destination inside or outside the cell [19]. Kex2 is a Golgi-resident, Ca^2+^-dependent, subtilisin-like, serine protease required for proprotein processing, a critical step during protein maturation before exiting from the Golgi complex [20]. It works in the *trans*-Golgi network, cycling between Golgi vesicles and late endosomal compartments [21]. The specificity of this protease is limited, hydrolyzing the peptide bond at the carboxyl side of Lysine–Arginine, and Arginine–Arginine sequences [22].

In *C. albicans*, loss of Kex2 affected the yeast morphology, generating larger cells that tended to form aggregates [23]. In addition, the *kex2*Δ null mutant did not undergo dimorphism when stimulated with albumin or incubation at 37 °C [23]. These mutant cells showed defects in virulence when analyzed in both mice and *Galleria mellonella* larvae [24,25,26]. This phenotype is likely explained by the lack of processing of aspartyl proteinase 2 and candidalysin, virulence factors that contribute to tissue damage [23,27]. Regarding the relevance of Kex2 during the *C. albicans*–immune cell interaction, the current information is limited. The loss of Kex2 did not affect the ability of murine macrophages to phagocyte yeast cells, but the mutant cells were not able to escape from the macrophages, even after 24 h of interaction, suggesting Kex2 is required for the lysis of macrophages [24]. There were also no defects reported in the ability of the *kex2*Δ null mutant to stimulate TNFα production by human peripheral blood mononuclear cells (PBMCs) [25].

Here, we aimed to explore whether this pleiotropic phenotype of the *kex2*Δ null mutant cells could also affect cell wall composition and organization and therefore, the interaction with innate immune cells.

## 2. Materials and Methods

### 2.1. Strains and Culture Conditions

The strains used in this study are shown in Table 1. The Ura + strain NGY152 [28], derived from strain SC5314, is referred to here as the wild-type (WT) strain. The cells were maintained at 28 °C in Sabouraud medium (1% [*w*/*v*] meat and casein peptone, 4% [*w*/*v*] glucose). When required, solid medium, 2% (*w*/*v*) agar was included in the Sabouraud medium. To propagate cells, an aliquot of 500 μL from an overnight culture was used to inoculate 50 mL of fresh Sabouraud broth and incubated at 28 °C with reciprocal shaking at 120 rpm, until mid-log growth phase was reached (typically 5 h). To induce expression of *HEX1*, cells were incubated in SC medium (0.7% [*w*/*v*] YNB with amino acids, 25 mM glucosamine) for 24 h at 28 °C [29]. For heat inactivation, the cells were incubated at 56 °C for 60 min. The loss of cell viability was confirmed by the absence of fungal growth in Sabouraud solid medium at 28 °C for 48 h.

### 2.2. Generation of a Ura + kex2Δ Null Mutant and the Re-Integrant Control Strain

The *kex*2Δ null mutant strain was previously generated [26], and is a Ura- derived mutant from CAI-4 strain. This strain was transformed with the StuI-linearized plasmid CIp10 to restore *URA3* at the *RPS1* locus, as described [30]. To generate the re-integrant control strain, the *KEX2* open reading frame (systematic name C1_08990C_A at www.candidagenome.org), plus ~1000 bp upstream and ~650 bp downstream were amplified by PCR using the primer pair 5′-GCGGCCGCAAAGTGTATAATTGAGGATGATTCGG and 5′-GCGGCCGCGATGCTATGTCGTAGAAATGCAGTA (underlined sequences indicate artificial NotI sites included in the primers). The PCR product was cloned into NotI sites of the CIp10 plasmid [30], generating CIp10-*KEX2*. The Ura-null mutant strain was transformed with StuI-linearized CIp10-*KEX2*, and insertion into the *RPS1* locus was confirmed by PCR.

### 2.3. Analysis of Cell Wall Composition

Yeast cells were propagated as described, harvested by centrifugation, and disrupted in a Precellys 24 homogenizer (Bertin, Montigny-le-Bretonneux, France) with eight cycles of 90 sec at 6000 rpm with resting periods on ice between cycles. Then, the cell homogenate was centrifuged, the pellet saved, washed with 1 M NaCl, and intracellular proteins released from the walls by treatment with 2% (*w*/*v*) SDS, 0.3 M β-mercaptoethanol, and 1 mM EDTA, as described elsewhere [31]. Cell walls were acid hydrolyzed with 2 M trifluoroacetic acid and analyzed by high-performance anion-exchange chromatography with pulsed amperometric detection (HPAEC-PAD) with a Dionex system (Thermo Fisher Scientific, Waltham, MA, USA) equipped with a CarboPac PA-1 column [31]. Samples were eluted with an isocratic gradient of 3.2 mM NaOH, as reported [32]. For quantification of cell wall proteins, cell walls were lyophilized and hydrolyzed with 1 N NaOH, as reported [31], and analyzed using the DC^TM^ Protein Assay (Bio-Rad, Hercules, CA, USA). To remove *N*-linked mannans from the cell wall, yeast cells were incubated for 20 h at 37 °C with 25 U of endoglycosidase H (New England Biolabs, Ipswich, MA, USA) in 100 mM sodium acetate, pH 5.2 [33], while *O*-linked mannans were trimmed by resuspending cells in 20 mL 1 N NaOH and gently shaken for 20 h at room temperature [34]. In both cases, cells were pelleted by centrifuging, the supernatants collected, the pH neutralized, lyophilized, and used to quantify the sugar content with the phenol-sulfuric acid method [35,36].

### 2.4. Alcian Blue Binding Assays

The cell wall phosphomannan content was determined by the ability of cells to bind the cationic dye Alcian blue. Cells grown at the exponential phase were pelleted by centrifugation, washed twice with deionized water, had their cell concentration adjusted to O.D._600nm_ = 0.2, aliquots of 1 mL were pelleted, and cells were resuspended in 1 mL of 30 μg/mL Alcian Blue (Sigma-Aldrich, St. Louis, MI, USA). After 10 min of incubation at room temperature, cells were pelleted and the O.D._620nm_ of supernatants was determined. The Alcian Blue bound to cells was calculated as previously described [37].

### 2.5. Fluorochrome Staining

For chitin labeling, cells were stained with 1 mg/mL fluorescein isothiocyanate-wheat germ agglutinin conjugate (WGA-FITC; Sigma) [38]. For β-1,3-glucan labeling, cells were incubated with 5 μg/mL IgG Fc-Dectin-1 chimera for 1 h at room temperature and protected from light, followed by incubation with 1 μg/mL donkey anti-Fc IgG-FITC for 30 min at room temperature [39,40]. Then, cells were washed twice with PBS and examined by fluorescence microscopy using a Zeiss Axioscope-40 microscope and an Axiocam MRc camera. The fluorescence quantification of 300 cells was calculated using Adobe Photoshop CS3 and the next formula: [(total of green pixels-background green pixels) × 100]/total pixels [33].

### 2.6. Cell Wall Porosity Assay

The wall porosity was calculated by the relative porosity of polycations as previously described [41]. Briefly, overnight-grown cells were inoculated into fresh Sabouraud broth, incubated for 5 h at 28 °C and 120 rpm, and washed twice with PBS. Aliquots containing 1 × 10^8^ cells were pelleted and suspended in either 10 mM Tris-HCl, pH 7.4 (buffer A), buffer A plus 30 μg/mL poly-l-lysine (MW 30–70 kDa, Sigma) or buffer A plus 30 μg/mL DEAE-dextran (MW 500 kDa, Sigma) and incubated for 30 min at 28 °C with reciprocal shaking at 1200 rpm. Then, cells were centrifuged, supernatants collected, and used to measure the O.D._260nm._ The relative cell wall porosity to DEAE-dextran was calculated as described, using the porosity to poly-l-lysine for data normalization [41].

### 2.7. Susceptibility to Cell Wall Perturbing Agents

*C. albicans* strains were tested for susceptibility to cell wall perturbing agents using a microdilution method as described [42]. The yeasts were grown until they reached the exponential phase, washed with deionized water and adjusted at O.D._600 nm_ = 0.05, and seeded in a 96-well plate containing fresh Sabouraud medium, and doubling dilutions of the following perturbing agents: Congo red (Sigma, 400 μg/mL), calcofluor white (Sigma, 400 μg/mL), hygromycin B (Sigma, 300 μg/mL) and sodium dodecyl sulfate (SDS) (BioRad, 0.1%, *w*/*w*). The plates were incubated at 28 °C for 24 h and gently shaken, and the O.D._600 nm_ measured. As controls, mock wells without the perturbing agents were included. Growth data were normalized as the percentage of those generated with the mock wells.

### 2.8. Electrophoretic Mobility Shift Assays of Hex1

*C. albicans* cells were grown in SC medium for 24 h at 28 °C with shaking, collected by centrifuging, resuspended in 10 mM Tris-HCl, pH 6.8, and mechanically broken with a Precellys 24 homogenizer, as described above. Then, the homogenate was centrifuged for 10 min at 17,000× *g* and the supernatant was saved. Samples containing 100 μg of total protein were loaded onto a 6% PAGE gel and run for 3 h at 70 V under native conditions. The β-*N*-acetylhexosaminidase activity was determined by incubating with 0.4 mM 4-methylumbelliferyl N-acetyl-β-d-glucosamine (Sigma) in 0.1 M citrate-KOH buffer, pH 4.5 for 30 min at 37 °C. The enzyme activity was revealed by exposing the gels to UV light [43]. In some experiments, the cell homogenates were *N*-linked deglycosylated by incubating the samples for 20 h at 37 °C with 25 U of endoglycosidase H, prior to the electrophoretic separation [31].

### 2.9. Antifungal Drug Susceptibility Assays

The susceptibility to antifungal drugs was performed as described in the M27-A3 yeast microdilution test, standardized by the CLSI [44]. For the water-soluble antifungal drugs, a stock solution of 64 μg/mL was prepared using RPMI 1640 as diluent. For amphotericin B, a stock solution of 16 μg/mL was prepared using DMSO as diluent. In all cases, 10-fold dilutions series were prepared for incubation with fungal cells. A 5 h culture of *C. albicans* was washed with PBS and the O.D._530 nm_ adjusted to 0.5. Then, a 1:1000 dilution was prepared with RPMI 1640 medium (supplemented with glutamine and without sodium bicarbonate, buffered with 0.164 M morpholino propanesulfonic acid, adjusted to pH 7 and with 0.2% (*w*/*v*) glucose) and this dilution was used to inoculate 100 μL in flat-bottom 96-well plates. As controls, wells only with the medium were included. The antifungal dilutions were included in aliquots of 100 µL per well and plates were incubated at 37 °C for 24 h and gently shaken. Then, the absorbance of the plates was measured at 490 nm and 530 nm with a Multiskan™ FC microplate photometer (Thermo).

### 2.10. Protoplast Generation and Differential Centrifugation

Sabouraud-grown cells were resuspended at an O.D._600nm_ 2.5 in 50 mM Tris-HCl buffer, pH 7.5, 1 M sorbitol, 0.8 M KCl, and 10 mM MgSO_4_, lyticase 0.25 mg/mL (Sigma) and 15 mM β-mercaptoethanol, and incubated at 37 °C for 30 min [45]. Under these conditions, the protoplast generation was calculated in 98% of the cell population. Protoplasts were washed twice with 50 mM Tris-HCl buffer, pH 7.5, 1 M sorbitol, 0.8 M KCl, and 10 mM MgSO_4_, and resuspended in 10 mM phosphate buffer, pH 6.0 added with 3.0 mM MgCl_2_. Cells were lysed in a Potter-Elvehjem and pestle (12 strokes), centrifuged at 1000× *g* for 10 min, the supernatant saved and loaded on a continuous 10–65% sucrose (*w*/*w*) density gradient and centrifuged at 232,000× *g* for 4 h at 4 °C, using a VTi 50 rotor (Beckman Coulter, Brea, CA, USA) [45]. Gradients were fractionated in 1 mL aliquots.

### 2.11. Enzyme Activity Assays

The α-mannosidase activity was determined as previously described [46]. Aliquots containing 100 µg of total protein in 50 mM MES-Tris buffer, pH 6.0 were mixed with 40 µM 4-methylumbelliferyl α-d-mannopyranoside (Sigma) incubated at 37 °C for 30 min. The reaction was stopped by adding 50 mM glycine-NaOH buffer, pH 11, and the fluorescence of released 4-methylumbelliferone was quantified in a Perkin-Elmer LS-5B luminescence spectrofluorometer, with excitation and emission set at 350 nm and 440 nm, respectively. The activity was expressed as nmoles of 4-methylumbelliferone generated per min. The α-mannosyltransferase activity was determined as described previously [34]. Briefly, aliquots of 100 µg protein suspended in 50 mM Tris-HCl, pH 7.2 were mixed with 10 mM MnCl_2_, 0.76 mM GDP-[^14^C] mannose (0.02 µCi; specific activity 273 mCi/mmol), 50 mM acceptor molecule, and incubated for 60 min at 30 °C. Then, reactions were passed through a 0.4 mL Dowex 1-X2 anion exchange resin to remove the unincorporated radioactivity, and the eluted material used to quantify the radiation incorporated to the acceptors [34]. The acceptors used were α-1,2-mannobiose, α-1,3-mannobiose, or α-1,6-mannobiose (all from Sigma).

### 2.12. Ethics Statement

The use of human primary cells in this study was approved by the Ethics Committee of Universidad de Guanajuato (permission 17082011). Human cells were collected from healthy adult volunteers after information about the study was disclosed and written informed consent was obtained.

### 2.13. Stimulation of Cytokine Production by Human PBMCs

Human PBMCs from venous blood were isolated using Ficoll-Histopaque-1077 (Sigma) and gradient centrifugation as described [33]. The PBMC-yeast interactions were carried out in round-bottom 96-well plates containing aliquots of 100 μL of 5 × 10^6^ PBMCs/mL in RPMI 1640 Dutch modification (added with 2 mM glutamine, 0.1 mM pyruvate, and 0.05 mg/mL gentamycin; all reagents from Sigma), and 100 μL of 1 × 10^6^ yeast cells/mL. The interactions were incubated for 24 h at 37 °C with 5% (*v*/*v*) CO_2_, plates were centrifuged for 10 min at 874× *g* and 4 °C; the supernatants were recollected and kept at −20 °C until used [36]. The concentration of TNF-α, IL-6, and IL-10 was quantified by ELISA using the kit ABTS ELISA Development from Peprotech (Rocky Hill, NJ, USA). Mock reactions where the fungal cells were not added to the interactions were included as controls. In some experiments, PBMCs were pre-incubated for 1 h at 37 °C with 200 μg/mL laminarin (Sigma) before interaction with fungal cells.

### 2.14. Differentiation of Human PBMCs to Macrophages

After PBMCs isolation, aliquots of 1 mL containing 2 × 10^7^ cells in RPMI supplemented with 1% (*v*/*v*) penicillin-streptomycin solution (Sigma) were placed in flat-bottom 24-well plates, and incubated for 2 h at 37 °C, 5% (*v*/*v*) CO_2_. Non-adherent cells were removed gently with the medium and adherent cells were washed with PBS at 37°C. Aliquots of 1 mL of X-VIVO 15 serum-free medium (Lonza) supplemented with 1% (*v*/*v*) penicillin-streptomycin solution and 10 ng/mL recombinant human granulocyte-macrophage colony-stimulating factor (Sigma) were added into each well and incubated for 7–8 days at 37 °C, 5% (*v*/*v*) CO_2_, with fresh medium exchanged every 3 days [33]. After 8 days of incubation, the supernatant was removed and cells were used for cytokine stimulation, as described above, or to assess the ability to uptake *C. albicans* cells. The macrophage–yeast ratio was 1:5.

### 2.15. Analysis of C. albicans Phagocytosis by Human PBMC-Derived Macrophages

Freshly grown yeast cells adjusted to a concentration of 2 × 10^7^ cell/mL were washed twice with PBS and labeled with 1 mg/mL Acridine orange (Sigma) as described [47]. The excess of dye was removed by washing cells twice with PBS and resuspended at a cell concentration of 3 × 10^7^ yeast cells/mL. Interactions were performed in aliquots of 800 μL of DMEM (Sigma), in 6-well plates with a macrophage–yeast ratio of 1:6 [36]. Plates were incubated for 2 h at 37 °C and 5% (*v*/*v*) CO_2_. Then, the macrophages were washed twice with cold PBS and suspended in 1.25 mg/mL Trypan blue as an external fluorescence quencher [47]. Samples were analyzed by flow cytometry in a MoFlo XDP system (Beckman Coulter) collecting 50,000 events gated for macrophage cells. Fluorescent signals were obtained using the FL1 (green) and FL3 (red) channels previously compensated with macrophage and yeast cells without labeling. Phagocytosis of yeast-like cells was analyzed from counted events in green (early stage of the phagocytosis process), in both green and red (intermediate stage of the phagocytosis process), and red (cells within acidified phagolysosomes, regarded as a late stage of the phagocytosis process) [36,48].

### 2.16. G. mellonella Survival Assays

Infection and killing assays of wax moth larvae were conducted as reported [33,35,48]. The last left pro-leg of larvae was disinfected with 70% (*v*/*v*) ethanol, and 2 × 10^7^ yeast cells contained in 10 μL of PBS were injected with a Hamilton syringe and a 26-gauge needle. Animals were kept at 28 °C and survival was monitored daily for 2 weeks [36]. Loss of irritability and body melanization were taken as signs of animal death. For fungal burden determination, larvae were injected with 2 × 10^7^ yeast cells as indicated above and incubated for 24 h at 28 °C. Both survival and dead animals were decapitated, the hemolymph collected and used to calculate the colony-forming units (CFUs), by serial dilutions of the hemolymph and incubation on Sabouraud plates at 28 °C for 48 h. Each experimental group contained 30 larvae, including a control group injected only with sterile PBS.

### 2.17. Cytotoxicity Assays in Hemocytes of G. mellonella

To determine the liberation of the enzyme lactate dehydrogenase, 20 larvae were injected in each experiment with yeast cells from the different strains under analysis. Larvae were injected as described to determine fungal burden, decapitated, and 10 μL of hemolymph was recovered per animal and suspended in PBS added with 3.8% (*w*/*v*) sodium citrate. Samples were analyzed with the Pierce LDH Cytotoxicity Assay (Thermo), according to the manufacturer’s instructions.

### 2.18. Statistical Analysis

Statistical analysis was performed using the GraphPad Prism 6 software. The effect of cell wall perturbing agents on the fungal growth was analyzed by two-way ANOVA. Stimulation of cytokines and phagocytosis by human cells were carried out in duplicate with samples from eight healthy donors, whereas the rest of the in vitro experiments were performed at least thrice in duplicate. The cytokine profiles, phagocytosis, lactate dehydrogenase levels, and CFU quantification were analyzed with the Mann-Whitney U test. Survival experiments using larvae from *G. mellonella* were performed with a total of 30 larvae per group, and data were analyzed using the log-rank test and are reported in Kaplan–Meier survival curves. Other results were analyzed with the parametric *T*-test. The statistical significance in all cases was set at *P* < 0.05. All data are represented as mean and standard error (SD).

## 3. Results

### 3.1. Generation of kex2Δ Null Mutant Strains with URA3 Integrated into the Neutral Locus RPS1

The *KEX2* gene has been previously disrupted in *C. albicans* by both the Ura blaster and mini-Ura blaster techniques [23,26], using as a genetic background an *ura3*Δ null mutant strain and replacement of *KEX2* open reading frame with *URA3*. In these mutant strains, *URA3* was either recycled, generating an Ura^-^ strain, or left in the *KEX2* locus [23,26]. Since the ectopic expression of *URA3,* or the absence of this gene, affects the *C. albicans* phenotype, including virulence [28], we decided to generate a strain with the *URA3* gene located in the neutral locus *RPS1*. It was previously demonstrated that *RPS1* is haplosufficient, i.e., the loss of one allele has minimal impact on the *C. albicans* phenotype [30]. Moreover, the *URA3* expression and enzyme activity of the encoding product are similar in both the native and *RPS1* loci [28]. Thus a popular strategy to overcome the pleiotropic effect associated with absence or ectopic expression of *URA3* is the insertion of this gene into *RPS1* [28]. The previously generated *kex*2Δ null mutant [26] was transformed with the linearized vector CIp10, generating strain HMY209 (see Table 1). In addition, to truly associate the phenotypical changes of this mutant to the disruption of *KEX2*, we generated a reintegrant control strain transforming the *kex*2Δ null mutant [26] with the linearized construction CIp10-*KEX2*, generating strain HMY210 (see Table 1). Thus, the two strains have restored the *URA3* in the same locus, allowing an unbiased comparison of phenotypes. Strain NGY152 [28] was used as a WT strain, as it also contains *URA3* placed into the *RPS1* locus.

**Figure 1:**
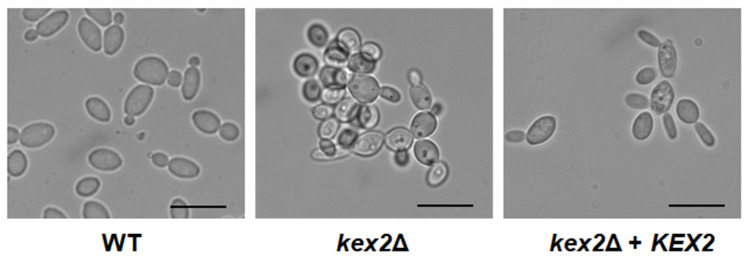
Cell morphology of *Candida albicans kex2*Δ null mutant. Cells were grown at 28 °C for 15 h in Sabouraud medium, demonstrating cell aggregates in the null mutant strain. Scale bar, 10 µm. Strains used are NGY152 (WT), HMY209 (*kex2*Δ), and HMY210 (*kex2*Δ + *KEX2*).

The *kex2*∆ null mutant showed defects in the cell morphology, displaying a clumpy phenotype that was absent in the reintegrant control and WT strains (Figure 1). As previously reported [23], the strain failed to undergo dimorphism when incubated in RPMI medium at 37 °C. When cells were grown in Sabouraud broth, the three strains showed similar doubling times (1.39 ± 0.4 h, 1.41 ± 0.5 h, and 1.40 ± 0.4 h for the WT, *kex2*∆, and reintegrant control strain, respectively; *P* = 0.95).

### 3.2. Loss of Kex2 Affects the C. albicans Cell Wall and Protein Glycosylation Pathways

To analyze the relevance of Kex2 in the *C. albicans* cell wall composition, walls were isolated, cleansed, acid-hydrolyzed, and analyzed by HPAEC-PAD in a Dionex system. The chitin, glucan, and mannan content was estimated by quantifying the basic monosaccharides of these polysaccharides, *N*-acetylglucosamine, glucose, and mannose, respectively [31,38,49,50]. The chitin, glucan, and mannan content in the cell wall of the WT control strain was similar to that previously reported for *C. albicans* [31,38,49,50], with a modest content of chitin and a large amount of glucan, followed by mannan (Table 2). The *kex2*Δ null mutant showed about seven-fold more chitin content than the WT control strain, a reduction of about 50% in the mannan content, and a small but significant increment in the glucan level (Table 2). The reintegrant control strain showed levels of chitin, mannan, and glucans similar to those found in the WT control cells (Table 2). The wall porosity to DEAE-dextran and phosphomannan content are parameters that have been previously associated with defects in the synthesis of mannans [31,33,35,36,49,51,52]. The phosphomannan content was reduced in about 50% in the null mutant strain, while the cell wall porosity showed a two-fold increment (Table 2). Both the WT and reintegrant control strains showed similar levels of phosphomannan and wall porosity (Table 2). Contrary to the reduction in the mannan content showed by the *kex2*Δ null mutant, the cell wall protein levels were increased in this strain (Table 2). Again, the WT and reintegrant control strains showed similar protein content in the cell wall (Table 2).

**Figure 2:**
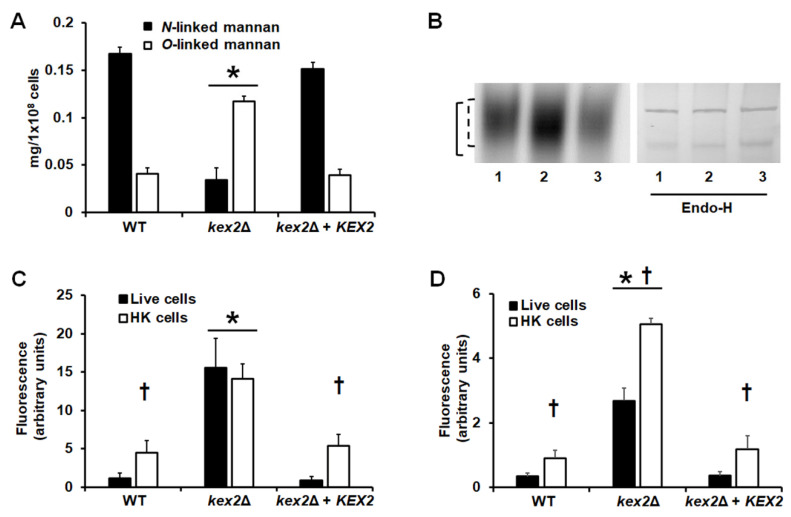
Analysis of cell wall components in the of *Candida albicans kex2*Δ null mutant and control strains. (**A**), cells were deglycosylated by incubating with endoglycosidase H (endo-H) or sodium hydroxide, to remove *N*-linked mannans or *O*-linked mannans, respectively, the hydrolyzed oligosaccharides were quantified as described in Materials and Methods. (**B**), Zymogram to reveal Hex1 activity. The dashed line indicates the electrophoretic mobility of the enzyme from both WT and reintegrant control strain, while on the continuous line is the mobility of the protein isolated from the *kex2*Δ null mutant. Endo-H refers to samples treated with endoglycosidase H. The height of both gels has been modified to make them shorter. (**C**), Cell wall chitin staining. Live or heat-killed (HK) yeast cells were labeled with fluorescein isothiocyanate-wheat germ agglutinin conjugate that binds chitin, as described in Materials and Methods, inspected under fluorescence microscopy, and the fluorescence associated to 300 individual cells recorded. (**D**), Cell wall β-1,3-glucan staining. Similar to panel (**C**), but cells were stained with IgG Fc-Dectin-1 chimera that binds β-1,3-glucan. Data are means ± SD of three independent experiments performed in duplicates. Strains used are NGY152 (WT, lane 1), HMY209 (*kex2*Δ, lane 2), and HMY210 (*kex2*Δ + *KEX2*, lane 3). * *P* < 0.05, when compared with either the WT or reintegrant control cells. † *P* < 0.05, when compared with live cells.

To further explore these changes in the cell wall, we quantified the content of *N*-linked and *O*-linked mannans, by deglycosylating the cell surface with either endoglycosidase H (endo H) or β-elimination [10,35,36]. The WT strain showed more *N*-linked mannan content than *O*-linked mannans, which is in agreement with previous reports [29,31,42,53] (Figure 2A). However, the *kex2*Δ null mutant showed a reduction in the *N*-linked mannan content and an increment in the *O*-linked mannan levels (Figure 2). The *KEX2* reintegration into the null mutant restored the content of both kinds of mannans to levels similar to those found in the WT strain (Figure 2A). The *C. albicans* Hex1, a β-*N*-acetylhexosaminidase, is a highly *N*-linked mannosylated protein that has been previously used to show defects in the *N*-linked mannosylation pathway [29,31,49]. Therefore, we performed non-denaturing protein electrophoresis followed by an in situ zymogram with a fluorogenic substrate [43]. The protein from the *kex2*Δ null mutant, but not that from the WT or the reintegrant control strains, had increased electrophoretic mobility, which suggested the presence of shorter *N*-linked mannans (Figure 2B). This was confirmed by performing the electrophoretic shift assay with samples previously deglycosylated with endo-H, where the proteins from the three strains had a similar migration profile (Figure 2B). Therefore, the *kex2*Δ null mutant has defects in the mannosylation pathways.

Next, we analyzed the distribution of chitin and β-1,3-glucan within the cell wall, labeling cells with WGA-FITC and an IgG Fc-Dectin-1 chimera, respectively [38,39,40]. The labeling of chitin and β-1,3-glucan in the WT and reintegrant control strains was localized mainly in the budding scars and the level of fluorescence associated with the cells was minimal (Figure 2C,D), suggesting most of these polysaccharides are not exposed on the cell surface and are therefore inaccessible to bind these lectins. However, the null mutant strain bound higher levels of both lectins, suggesting both chitin and β-1,3-glucan are exposed on the wall surface (Figure 2C,2D). It has been previously reported that yeast killing by heat artificially exposed inner wall components in the cell wall surface [54]. When these experiments were repeated with heat-killed (HK) cells, chitin labeling was higher in both the WT and reintegrant control cells (Figure 2C), confirming this previous observation. However, no significant increment in chitin labeling was observed in the HK *kex2*Δ null mutant strain (Figure 2C), suggesting that the whole content of chitin is exposed at the surface of the cell wall. For the case of β-1,3-glucan, heat killing affected the labeling of this wall polysaccharide in the wall of the three strains under study, suggesting that not all the β-1,3-glucan is exposed on the surface of the *kex2*Δ null mutant strain (Figure 2D).

**Figure 3:**
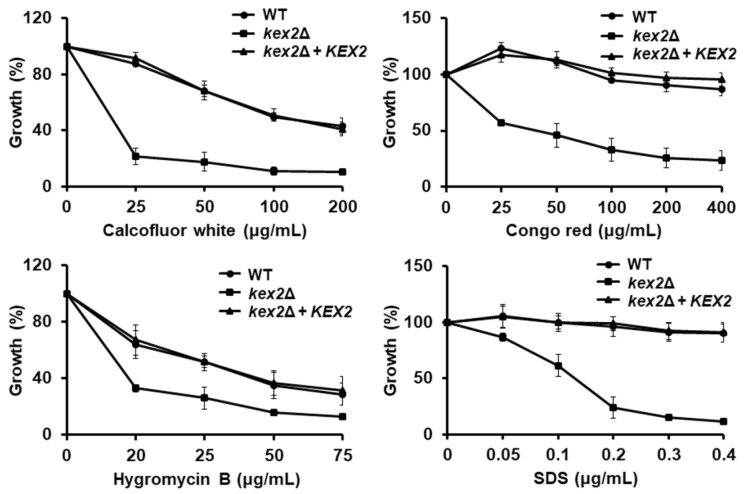
Susceptibility to calcofluor white, Congo red, hygromycin B, and sodium dodecyl sulfate in the *Candida albicans kex2*Δ null mutant and control strains. Cells were incubated in Sabouraud broth supplemented with different concentrations of calcofluor white, Congo red, Hygromycin B, or sodium dodecyl sulfate (SDS) and cell growth was determined after incubation for 24 h at 30 °C. For normalization, growth results are shown as a percentage of those obtained with the same strain grown in the absence of any perturbing agent. Data are means ± SD of three independent experiments performed in duplicates. (*P* < 0.01 when compared by two-way ANOVA). Strains used are NGY152 (WT), HMY209 (*kex2*Δ) and HMY210 (*kex2*Δ + *KEX*2).

The defects in *C. albicans* cell wall mannosylation are often associated with increased susceptibility to cell wall perturbing agents [29,31,42,49,53], such as Congo red, calcofluor white, hygromycin B, and SDS. The β-1,3-glucan structure is affected when cells are incubated with Congo red [55]; while calcofluor white binds and disturbs cell wall chitin, and the susceptibility to hygromycin B tends to increase when cells have defects in the protein glycosylation pathways [38,56]. In addition, the susceptibility of SDS increases when the cells show defects in either the cell wall or the plasma membrane [29,42]. The *kex2*Δ null mutant cells showed increased susceptibility to these four agents; while the WT and reintegrant control strains displayed a similar ability to grow under the presence of these compounds (Figure 3). Moreover, loss of Kex2 affected the *C. albicans* susceptibility to antifungal drugs, as the null mutant strain showed a minimal inhibitory concentration of 0.12 and 0.12 µg/mL for fluconazole and itraconazole, respectively, and these were lower than those found in the WT and re-integrant control strains (2.0 and 1.2 µg/mL for fluconazole and itraconazole in the WT strain; 1.1 and 2.0 µg/mL for fluconazole and itraconazole in the reintegrant control strains; and *P* < 0.05 when compared to the values of the null mutant with the control strains). Interestingly though, when the susceptibility to amphotericin B was tested, the three strains showed the same minimal inhibitory concentration of 0.25 µg/mL.

### 3.3. Loss of Kex2 Affects the Localization and the Activity of Mannosyltransferases

We have previously demonstrated that the α-1,2-mannosidase activity in *C. albicans* is localized in the cytosolic compartment, the endoplasmic reticulum, and the Golgi complex [45]. The first activity is a soluble isoform generated by the proteolytic processing of a membrane-bound α-1,2-mannosidase by Kex2 [45]. Upon protoplast generation and fractionation of subcellular compartments by centrifuging in a 10–65% sucrose gradient, the distribution of mannosidase activity in the WT strain was associated with three peaks with calculated densities of 1.036 ± 0.008 g/cm^3^, 1.133 ± 0.010 g/cm^3^, and 1.186 ± 0.009 g/cm^3^, which correspond to those densities previously established for the cytosolic compartment, Golgi complex, and endoplasmic reticulum, respectively [45]. The distribution of the enzyme activity was 19.7%, 27.0%, and 53.3% in the cytosolic compartment, Golgi complex, and endoplasmic reticulum, respectively (Table 3), which is in agreement with our previous observations [45]. A similar enzyme distribution was observed in the reintegrant control strain (Table 3). As expected [45], the cytosolic activity of α-1,2-mannosidase was absent in the *kex2*Δ null mutant strain, with no defects in the enzyme distribution in the Golgi complex and the endoplasmic reticulum (Table 3). Since these data indicated an appropriated separation of at least these three compartments by our differential centrifugation, we next assessed the distribution of mannosyltransferase activities associated with the Golgi complex. We can differentiate the Golgi-resident and endoplasmic reticulum-resident mannosyltransferases because the former exclusively use GDP-mannose as a sugar donor, while the latter utilize mannose residues attached to dolichol phosphate [5,12,34]. Using GDP-mannose as a sugar donor, we found that in the three strains under analysis, trace amounts of α-1,2-mannosyltransferase activity were found in the cytoplasmic compartment, whereas the activity of α-1,3- and α-1,6-mannosyltransferases was under the detection limit in the same compartment (Table 3). Similarly, no changes in the distribution and amount of the α-1,3-mannosyltransferase activity were observed in the Golgi complex and endoplasmic reticulum of the WT, null mutant, and reintegrant control strains (Table 3). However, in the case of the α-1,2-mannosyltransferase activity, this was significantly higher in the Golgi complex of the *kex2*Δ null mutant cells, with no changes in the activity associated with the endoplasmic reticulum (Table 3). In the case of the α-1,6-mannosyltransferase activity, it was significantly reduced in the Golgi complex of the null strain, again, with no changes in the activity levels found in the endoplasmic reticulum (Table 3). The activity of these two enzymes was similar for the WT and reintegrant control strains in the three intracellular compartments analyzed (Table 3). Therefore, loss of Kex2 affected the activity of Golgi-resident α-1,2- and α-1,6-mannosyltransferases.

### 3.4. The C. albicans kex2Δ Null Mutant Shows Abnormal Interactions with Human PMBCs and Monocyte-Derived Macrophages

**Figure 4:**
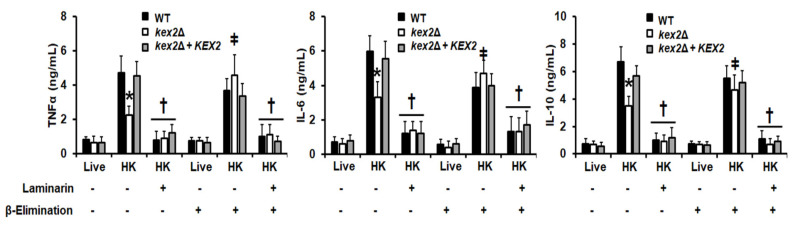
Cytokine production by human peripheral blood mononuclear cells stimulated with *Candida albicans kex2*Δ null mutant and control strains. Live or heat-killed (HK) yeast cells and human peripheral blood mononuclear cells were co-incubated for 24 h, the supernatant saved and used to quantify the levels of secreted TNFα, IL-6, and IL-10. Results (means ± SD) were obtained using samples from eight donors, each assayed in duplicate wells. * *P* < 0.05, when compared with live cells under the same treatment; † *P* < 0.05, when compared with HK cells without preincubation with laminarin; ǂ *P* < 0.05, when compared to cells without β-elimination treatment. Strains used are NGY152 (WT), HMY209 (*kex2*Δ), and HMY210 (*kex2*Δ + *KEX2*).

Next, to assess the relevance of Kex2 during the interaction of *C. albicans* with components of the innate immunity, we quantified the levels of cytokines produced by human PBMCs upon interaction with the fungal cells. As previously reported [10,11,13,31,49,54], live cells of the WT control strain stimulated low levels of TNF-α, IL-6, and IL-10 (Figure 4), and similar results were observed with the *kex2*Δ null mutant cells and the reintegrant control strain (Figure 4). One of the main *C. albicans*-associated molecular patterns that stimulate cytokine production is β-1,3-glucan, and as mentioned, most of it is masked by mannans [13,18,54]. Therefore, we exposed this wall component on the cell surface by heat inactivation and performed the cytokine stimulation assays with these cells. The HK WT and reintegrant control strains stimulated a similar and strong TNF-α, IL-6, and IL-10 production, which contrasted with the modest cytokine levels stimulated by the *kex2*Δ null mutant strain (Figure 4). Since the characterization of the cell wall of the null mutant showed an increment in the content of *O*-linked mannans we next explored the contribution of this wall component to the cytokine stimulation by the null mutant strain. Upon removal of *O*-linked mannans by β-elimination, cells were subjected again to interaction with the human cells, and results indicated no significant differences in the three cytokine levels stimulated by live or live + β-eliminated cells from the three strains under study (Figure 4). Even though HK + β-eliminated cells from the three strains stimulated the production of similar levels of TNF-α, IL-6, and IL-10, the cytokine levels stimulated by the *kex2*Δ null mutant cells were higher than those produced by the interaction of PBMCs with HK mutant cells, i.e., with cells containing *O*-linked mannans on the surface (Figure 4).

To explore the contribution of the β-1,3-glucan-dectin-1 signaling to the cytokine stimulation, we pre-incubated the human PBMCs with the antagonist of dectin-1, laminarin, and used these cells for cytokine stimulation. There were no changes in the three cytokine levels when live fungal cells from the three strains and laminarin-preincubated cells were used in the interactions but when HK or HK + β-eliminated cells were used there was a significant reduction in the TNF-α, IL-6, and IL-10 levels, indicating the production of these cytokines is associated to the activation of dectin-1 via engagement with β-1,3-glucan (Figure 4).

**Figure 5:**
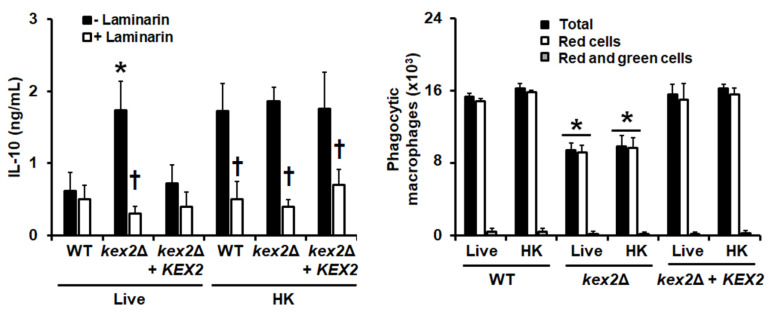
The interaction of *Candida albicans* with monocyte-derived macrophages is affected by the loss of Kex2. In the **left panel**, live or heat-killed (HK) yeast cells and human monocyte-derived macrophages were co-incubated for 2 h, the supernatant saved and used to quantify IL-10. In the **right panel**, before the cell–cell interaction, the yeast cells were labeled with 1 mg/mL Acridine orange and after the 2 h incubation, the human cells were analyzed by flow cytometry as described in the Materials and Methods section. Total refers to the total number of macrophages phagocytosing at least one yeast cell, red cells and red and green cells are regarded as yeast cells in the late and intermediate stages of the phagocytosis process, respectively. For both panels, Results (means ± SD) were obtained using samples from eight donors, each assayed in duplicate wells. * *P* < 0.05, when compared with WT or reintegrant control cells under the same treatment; † *P* < 0.05, when compared with cells without preincubation with laminarin. Strains used are NGY152 (WT), HMY209 (*kex2*Δ), and HMY210 (*kex2*Δ + *KEX2*).

We also explored the interaction of the *kex2*Δ null mutant with human monocyte-derived macrophages. Upon the fungus-immune cell interaction, the stimulation of the proinflammatory cytokines TNFα and IL-6 was similar for the null mutant and the control strains, regardless of whether they were live or HK cells. In the case of IL-10 production, live cells of the *kex2*Δ null mutant stimulated higher levels when compared to both the WT and reintegrant control strains (Figure 5). The use of HK cells did not change the IL-10 levels stimulated by the null mutant, but for the WT and reintegrant control strains both strains stimulated higher levels of this cytokine when compared to live cells (Figure 5). The cytokine production stimulated by the live mutant cells or the HK cells from the three strains was dependent on dectin-1 stimulation, as pre-incubation of monocyte-derived macrophages with laminarin significantly reduced the IL-10 production (Figure 5). When the ability of these immune cells to phagocyte the *kex2*Δ null mutant was analyzed, we found that a reduced amount of cells was phagocytosed by the monocyte-derived macrophages, and higher and similar numbers of WT and reintegrant control cells were phagocytosed by these primary immune cells (Figure 5). Our strategy to analyze the cell uptake by the immune cells allowed us to discriminate green yeast cells, green and red cells, and red cells, which are regarded as yeast in the early stage, intermediate stage, or in the late stage of the phagocytosis process [36,48]. In our system, for the three strains, most of the fungal cells were in the late stage of the phagocytosis process, i.e., inside acidified phagolysosomes, with a small proportion in the intermediate phase (Figure 5). The number of cells in the early stage of phagocytosis was marginal, with similar values to those obtained with human cells with no yeast cells included. Collectively, these data indicate that a loss of Kex2 affected the interaction of *C. albicans* with human PBMCs and monocyte-derived macrophages.

### 3.5. The C. albicans kex2Δ Null Mutant Shows Defects in the Ability to Kill G. mellonella Larvae

**Figure 6:**
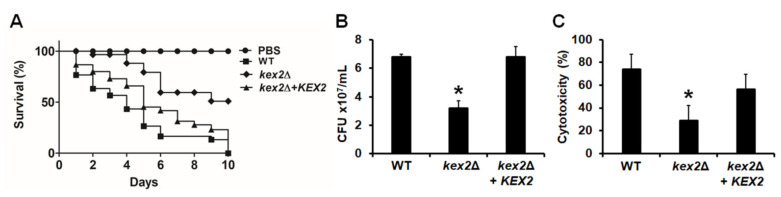
The *Candida albicans kex2*Δ null mutant strain display defects in the interaction with larvae of *Galleria mellonella*. In (**A**), groups of 30 larvae of *G. mellonella* were injected with cells from the three strains under study and the survival was monitored daily for ten days. As a control, one animal group was inoculated with the vehicle for cell dilution (PBS). In (**B**), the hemolymph was recovered from both life and killed animals, aliquots were plated on Sabouraud plates, incubated at 28 °C for 48 h, and the colony-forming units (CFU) determined. In (**C**), groups of 20 animals were inoculated with 2 × 10^7^ cells from the strains under analysis, incubated for 24 h at 28 °C, and then the animals were decapitated, 10 μL of hemolymph recovered per animal and used to quantify the amount of released lactate dehydrogenase. For (**B**) and (**C**), results are means ± SD. * *P* < 0.05, when compared with WT or reintegrant control cells. Strains used are NGY152 (WT), HMY209 (*kex2*Δ), and HMY210 (*kex2*Δ + *KEX2*).

The virulence of the *C. albicans kex2*Δ null mutant has been previously assessed in a mouse model of systemic candidiasis and in larvae of *G. mellonella* [24,26]. However, in both cases, the strains used contain *URA3* in the *KEX2* locus, and as mentioned, this ectopic *URA3* expression has been reported to affect cellular traits, including virulence [28]. Thus, we reevaluated this cellular trait with the strains generated here, where *URA3* is placed in the neutral *RPS1* locus [28], using *G. mellonella* larvae. When animals were inoculated with either the WT control strain or the reintegrant control cells, the whole animal population was killed by day 10 post-inoculation, and no significant differences were observed in the survival curves (*P* = 0.1). However, only 50% of larvae infected with the *kex2*Δ null mutant were killed at the same time of inspection, showing virulence attenuation (Figure 6A). The fungal burden in the hemolymph of inoculated animals was significantly lower in the animal group inoculated with the null mutant strain, while both the WT and reintegrant control strains showed higher and similar CFUs (Figure 6B). We also quantified the amount of released lactate dehydrogenase in the hemolymph, since the presence of this intracellular enzyme in the extracellular compartment has been associated with cytotoxicity [25,35]. The extracellular enzyme in the hemolymph of animals infected with the *kex2*Δ null mutant was significantly lower than those levels found in the animal groups inoculated with either the WT of reintegrant control strains (Figure 6C), suggesting less cell damage in animals infected with the null mutant strain. Collectively, these data indicate that the *C. albicans kex2*Δ null mutant strains showed defects in the ability to kill and damage the larvae of *G. mellonella*.

## 4. Discussion

Previous work has reported that *KEX2* disruption affected the host–fungus interaction, dimorphism, and expression of some virulence factors, such as aspartyl proteinases and candidalysin [23,24,25,26,27]. However, thus far no attention has been given to the cell wall of this mutant strain. Here, we found that the loss of Kex2 led to significant changes in the composition and organization of this cell structure. It has been previously suggested that the cell aggregation observed in mutant strains with defects in the mannosylation pathways could be due to the inability of cell separation because of the accumulation of chitin at the cell wall [29,31,42,49,52]. Since the *kex2*Δ null mutant strain showed both cell aggregation and accumulation of chitin in the cell wall, it is feasible to suggest that the clumpy phenotype is due to the increased chitin content in the cell wall. It was interesting to observe an increment in the protein levels at the cell wall of the null mutant strain, and a similar observation was reported for *C. albicans* mutants with defects in the *N*-linked mannosylation pathway [31]. It has been described that the canonical secretory pathway is not the only one involved in the transportation of proteins outside the *Candida* cell [57]. We hypothesize that this increment in the cell wall protein content in the *kex2*Δ null mutant strains is therefore due to the activation of noncanonical protein secretory pathways, as a compensatory mechanism to overcome the defect in the classical protein secretory pathway. Nonetheless, this remains to be confirmed.

It has been previously reported that defects in the mannosylation pathways led to the activation of the cell wall integrity pathway [29,31,42,58], and as a result, the chitin and β-glucan content are increased to compensate for the lack of a proper mannan layer. This is the most likely explanation behind the increased content of both structural polysaccharides in the *kex2*Δ cell wall and the increased susceptibility to both Congo red and calcofluor white. The high chitin content combined with the low mannan levels affected the normal localization of this polysaccharide, most of it now being at the cell surface. In the case of the β-1,3-glucan, part (but not all) of this polysaccharide was exposed at the cell surface, and this has significant implications during the *C. albicans*–immune cell interaction, as described in lines below.

The defect in the cell wall mannosylation was evidenced by quantifying the whole content of wall mannose, by the reduced content of phosphomannan, changes in the released mannans upon deglycosylation with endo H or β-elimination, and the increased electrophoretic mobility of Hex1, a secreted protein exclusively and highly *N*-linked mannosylated [29,31,49]. Moreover, this was further evidenced by the increased sensitivity to wall perturbing agents and antifungal drugs, as demonstrated to occur in other glycosylation pathway mutants of *C. albicans* and other *Candida* species [29,31,33,35,42,48,49,52,53]. Increased susceptibility to antifungal drugs has been also observed in cells with defects in the PMT gene family, whose members participate in the *O*-linked mannosylation pathway [59] and in mutant strains with changes in the cell wall glycosylphosphatidylinositol-anchored proteins [32]. In the case of amphotericin B, the null mutant and control strains showed the same drug susceptibility. It is possible to speculate that the action mode of amphotericin B, which relies on binding to plasma membrane ergosterol, is not modified by any of the phenotypical traits affected by the disruption of *KEX2*.

Our results clearly showed that upon *KEX2* disruption, some Golgi-resident mannosyltransferase activities were affected. The Och1 α-1,6-mannosyltransferase is one of the main enzymes that contribute to this enzyme activity in the Golgi complex [5,12,29]. Even though it has been regarded as an enzyme localized in the early Golgi complex, this enzyme is, in fact, a dynamic component of the organelle and is also found in the trans-Golgi network and even in the endoplasmic reticulum [60,61]. The trafficking, and therefore the localization, of Och1 in the early Golgi requires the conserved oligomeric Golgi complex 3 (Cog3) protein [62], and a global genetic analysis to establish interaction networks in *Saccharomyces cerevisiae* found that Cog3 and Kex2 positively interact [63]. Thus, our hypothesis to explain the reduction of α-1,6-mannosyltransferase activity is that this Cog3–Kex2 interaction is not taking place in the *C. albicans* null mutant strain, and as a consequence, this enzyme is mislocalized, most likely outside the compartments involved in the secretory pathway. Since this enzyme is key for the elaboration of the *N*-linked mannan outer chain [5,12,29], the *N*-linked mannosylation pathway will be affected, generating truncated oligosaccharides, as evidenced in the *kex2*Δ null mutant. To complement this hypothesis, positive genetic interactions have been reported between Kex2 and Mnn11 or Hoc1 [64], Golgi-resident proteins with putative α-1,6-mannosyltransferase activity in *C. albicans* [5,12]. Another possible explanation to our observation is that the endoplasmic reticulum glucosidases I and II positively interact with Kex2 [65], and since the loss of these proteins negatively affected the *C. albicans N*-linked mannosylation pathway, it is possible to offer a second and complementing explanation to connect the Kex2 disruption with the defect in the *N*-linked mannosylation pathway. The change in the size of the *N*-linked mannan outer chain could expose serine or threonine residues that are naturally hidden by these structures, and will now be susceptible to modification with *O*-linked mannans, increasing the content of this structures in the cell wall of the mutant strain. The α1,3-mannosyltransferase activity and Kex2 have been localized in different compartments of the *S. cerevisiae* Golgi complex [66], and this could explain the minimal effect of *C. albicans* Kex2 disruption on this enzyme activity.

In terms of the ability to stimulate cytokine production by the *kex2*Δ null mutant cells, it is possible to link some of the cell wall changes to the modification in the cytokine production by human PBMCs. It has been previously demonstrated that *O*-linked mannans have a minimal role in cytokine stimulation [10,13,49], and our results confirmed this observation, as the live *kex2*Δ null mutant strain showed increased content of this wall component, but no modified ability to stimulate PBMCs. The cytokine production stimulated by the HK mutant cells upon β-elimination leads us to propose that *O*-linked mannans are masking other more relevant molecular patterns for cytokine stimulation, which has been previously proposed in *Candida parapsilosis* and *Candida guilliermondii* [33,35]. As reported by other groups [14,51,67,68,69], the recognition of β-1,3-glucan by dectin-1 was a central interaction for the stimulation of cytokines by the *kex2*Δ null mutant, as the levels of TNFα, IL-6, and IL-10 were significantly reduced when the human PBMCs were blocked with laminarin. The sensing of β-1,3-glucan is also key and sufficient to establish the interaction between macrophages and *C. albicans* [70] and it has been reported that the IL-10 stimulation can be achieved by the sole activation of the dectin-1-dependent signaling pathway [71]. Since the *kex2*Δ null mutant has increased levels of β-1,3-glucan and this is partially exposed at the cell surface, this could account for the increased IL-10 production observed in the mutant cell-macrophage interaction. For the case of uptake by macrophages, additional signaling pathways are, however, required. Besides the sensing of β-1,3-glucan, macrophages require the recognition of fungal phosphomannan [72], which is lower in the mutant cell wall, negatively affecting the phagocytic process. Moreover, it was reported that *O*-linked mannans negatively affect the *C albicans* uptake by macrophages [72], and this wall component is more abundant in the cells where *KEX2* was disrupted. Our results contrast with previous studies, where the interaction of macrophages with these mutant cells was minimally affected [24]. These previous experiments were conducted with fungal cells where *URA3* was ectopically expressed, the immune cells were a murine cell line, and the approach to analyze the interaction was based on microscopy [24]. The sum of all, or part of these factors, is likely to account for the discrepancy of our results with those already reported. Finally, our results confirmed that virulence was affected upon disruption of *KEX2* [24,26], but contrasting with our previous study in *G. mellonella* where an avirulent phenotype was reported [26], here we only observed attenuation of the virulence, with 50% of the animal population killed by the mutant strain. These results stress the relevance to reintegrate *URA3* in a neutral locus to truly assess the contribution of the studied gene to the cellular phenotype, as reported [28].

In conclusion, the generation of a *kex2*Δ null mutant strain with *URA3* in the *RPS1* locus, along with a reintegrant control strain, allowed us to establish that *KEX2* disruption affected the *C. albicans* cell wall and the interaction with human PBMCs and monocyte-derived macrophages, and also confirmed the relevance of this gene for the *C. albicans* virulence.

## Figures and Tables

**Table 1:** *Candida albicans* strains used in this work.

Strain	Origin	Genotype	Reference
NGY152	Derived from CAI-4	*ura3*Δ-*iro1*Δ::*imm434*/*ura3*Δ-*iro1*Δ::*imm434*; *RPSI*/*rps1*Δ::CIp10	[28]
*kex2*Δ/*kex*2Δ	Derived from CAI-4	*ura3*Δ-*iro1*Δ::*imm434*/*ura3*Δ-*iro1*Δ::*imm434*; *kex2*Δ::dp1200/*kex2*Δ::dp1200	[26]
HMY209	Derived from *kex2*Δ/*kex*2Δ	As *kex2*Δ/*kex*2Δ, but *RPSI*/*rps1*Δ::CIp10	This work
HMY210	Derived from *kex2*Δ/*kex*2Δ	As *kex2*Δ/*kex*2Δ, but *RPSI*/*rps1*Δ:: CIp10-*KEX2*	This work

**Table 2:** Cell wall composition of *Candida albicans kex2*Δ and control strains.

Cell Wall Abundance				Phosphomannan Content (μg) ^a^	Porosity (%) ^b^	Protein Content (μg) ^c^
Strain	Chitin (%)	Mannan (%)	Glucan (%)			
WT (NGY152)	1.8 ± 0.5	43.7 ± 1.0	54.4 ± 0.7	95.7 ± 9.1	22.4 ± 7.9	62.4 ± 4.7
*kex2*Δ (HMY209)	8.8 ± 1.0 *	28.1 ± 0.5 *	63.0 ± 0.8 *	45.9 ± 15.2 *	70.5 ± 12.4 *	96.8 ± 10.0 *
*kex2*Δ + *KEX2* (HMY210)	1.9 ± 0.5	41.6 ± 0.5	56.5 ± 0.6	89.1 ± 12.5	27.1 ± 8.8	55.0 ± 4.0

^a^ µg of Alcian Blue bound/OD_600_ = 1.0, ^b^ Relative to DEAE-dextran, ^c^ μg per mg of cell wall, * *P* < 0.05, when compared to the WT or reintegrant strains.

**Table 3:** Mannosidase and mannosyltransferase activities in some subcellular compartments of *Candida albicans kex2*Δ null mutant and control strains.

Enzyme Activity		Strain		
		WT (NGY152)	*kex2*Δ (HMY209)	*kex2*Δ + *KEX2* (HMY210)
α-1,2-Mannosidase ^a^	Cytosolic compartment	6.9 ± 1.5	0.02 ± 0.01 *	6.3 ± 1.1
	Golgi complex	9.5 ± 1.3	8.9 ± 2.0	9.0 ± 1.8
	Endoplasmic reticulum	18.7 ± 1.9	20.7 ± 2.7	19.1 ± 1.6
α-1,2-Mannosyltransferase ^b^	Cytosolic compartment	5.0 × 10^2^ ± 2.0 × 10^2^	4.0 × 10^2^ ± 3.0 × 10^2^	3.0 × 10^2^ ± 2.0 × 10^2^
	Golgi complex	8.0 × 10^6^ ± 9.0 × 10^5^	14.0 × 10^6^ ± 1.1 ×10 *	9.0 × 10^6^ ± 1.2 × 10^6^
	Endoplasmic reticulum	6.0 × 10^3^ ± 1.5 × 10^3^	4.0 × 10^3^ ± 1.1 × 10^3^	2.0 × 10^3^ ± 1.5 × 10^3^
α-1,3-Mannosyltransferase ^b^	Cytosolic compartment	ND	ND	ND
	Golgi complex	3.0 × 10^6^ ± 1.0 × 10^6^	3.0 × 10^6^ ± 1.8 × 10^6^	2.0 × 10^6^ ± 0.8 × 10^6^
	Endoplasmic reticulum	8.0 × 10^2^ ± 2.4 × 10^2^	7.0 × 10^2^ ± 1.1 × 10^2^	9.0 × 10^2^ ± 3.5 × 10^2^
α-1,6-Mannosyltransferase ^b^	Cytosolic compartment	ND	ND	ND
	Golgi complex	9.0 × 10^5^ ± 1.8 × 10^5^	2.0 × 10^2^ ± 0.9 × 10^2^ *	7.0 × 10^5^ ± 2.4 × 10^5^
	Endoplasmic reticulum	3.0 × 10^2^ ± 0.6 × 10^2^	2.4 × 10^2^ ± 1.0 × 10^2^	2.6 × 10^2^ ± 0.9 × 10^2^

^a^ Expressed as nmoles of 4-methylumbelliferone generated per min per mg of protein, ^b^ Expressed as cpm per min per mg of protein, ND, No detected, * *P* < 0.05 when compared to either the WT or reintegrant control strains.
